# Synaptic Organizers in Alzheimer’s Disease: A Classification Based on Amyloid-β Sensitivity

**DOI:** 10.3389/fncel.2020.00281

**Published:** 2020-09-02

**Authors:** Alfred Kihoon Lee, Husam Khaled, Nicolas Chofflet, Hideto Takahashi

**Affiliations:** ^1^Synapse Development and Plasticity Research Unit, Institut de Recherches Cliniques de Montréal (IRCM), Montreal, QC, Canada; ^2^Integrated Program in Neuroscience, McGill University, Montreal, QC, Canada; ^3^Molecular Biology Program, Université de Montréal, Montréal, QC, Canada; ^4^Department of Medicine, Université de Montréal, Montreal, QC, Canada; ^5^Division of Experimental Medicine, McGill University, Montreal, QC, Canada

**Keywords:** Alzheimer’s disease, amyloid-β, synaptic organizers, neurexin, neuroligin, *in situ* binding assay, artificial synapse formation assay

## Abstract

Synaptic pathology is one of the major hallmarks observed from the early stage of Alzheimer’s disease (AD), leading to cognitive and memory impairment characteristic of AD patients. Synaptic connectivity and specificity are regulated by multiple trans-bindings between pre- and post-synaptic organizers, the complex of which exerts synaptogenic activity. Neurexins (NRXs) and Leukocyte common antigen-related receptor protein tyrosine phosphatases (LAR-RPTPs) are the major presynaptic organizers promoting synaptogenesis through their distinct binding to a wide array of postsynaptic organizers. Recent studies have shown that amyloid-β oligomers (AβOs), a major detrimental molecule in AD, interact with NRXs and neuroligin-1, an NRX-binding postsynaptic organizer, to cause synaptic impairment. On the other hand, LAR-RPTPs and their postsynaptic binding partners have no interaction with AβOs, and their synaptogenic activity is maintained even in the presence of AβOs. Here, we review the current evidence regarding the involvement of synaptic organizers in AD, with a focus on Aβ synaptic pathology, to propose a new classification where NRX-based and LAR-RPTP-based synaptic organizing complexes are classified into Aβ-sensitive and Aβ-insensitive synaptic organizers, respectively. We further discuss how their different Aβ sensitivity is involved in Aβ vulnerability and tolerance of synapses for exploring potential therapeutic approaches for AD.

## Introduction

Alzheimer’s disease (AD), the most common age-related neurodegenerative disease with progressive cognitive decline including memory loss, has seen a sharp increase in the number of cases and AD-related deaths over the past decades. Although some therapies are clinically applied to AD patients, at best they slightly delay the disease progression and temporarily improve some symptoms (Weller and Budson, 2018; Long and Holtzman, 2019). Thus, a deeper understanding of the mechanisms involved in AD development and progression is indispensable for establishing better treatments for this disease.

There are two major pathohistological hallmarks of the AD brain: extracellular senile plaques and intracellular neurofibrillary tangles (NFT), the major constituents of which are amyloid β (Aβ) peptides and hyper-phosphorylated tau proteins, respectively (Ballard et al., 2011; DeTure and Dickson, 2019). Aβ has been reported to be a key detrimental molecule that plays a major role in AD pathogenesis (Reiss et al., 2018). Aβ is produced from the cleavage of amyloid precursor protein (APP) by β- and γ-secretases (Haass and Selkoe, 2007; O’Brien and Wong, 2011), after which it is secreted to the extracellular space and forms oligomers. Aβ oligomers (AβOs) are thought to be toxic for neurons and their synaptic connections in AD patient brains (Haass and Selkoe, 2007; Sheng et al., 2012). Indeed, many *in vitro* studies using primary neuron cultures (Parodi et al., 2010; He et al., 2019), brain slices (Hsieh et al., 2006; Shankar et al., 2007; Li et al., 2011) and *in vivo* studies (Spires-Jones et al., 2007; Hong et al., 2016) using AD model mouse lines overproducing Aβ (e.g., J20 and Tg2576) have supported the toxic effects of AβOs by showing Aβ-induced synaptic loss, decreased presynaptic release probability and impaired postsynaptic long-term potentiation (LTP), which is synaptic plasticity depending on postsynaptic *N*-Methyl-*D*-aspartate-type glutamate receptor (NMDAR)-mediated pathways (Nicoll, 2017). According to previous studies, Aβ pathology seems to precede tau pathology and importantly to start even from preclinical AD stage (Jansen et al., 2015; Sasaguri et al., 2017; van der Kant et al., 2020). Furthermore, synapse loss is an early pathological feature of AD and one of the best correlates of cognitive impairment (Scheff and Price, 2003; Sheng et al., 2012). These suggest the importance of understanding the mechanism of Aβ synaptic pathology.

**Figure 1:**
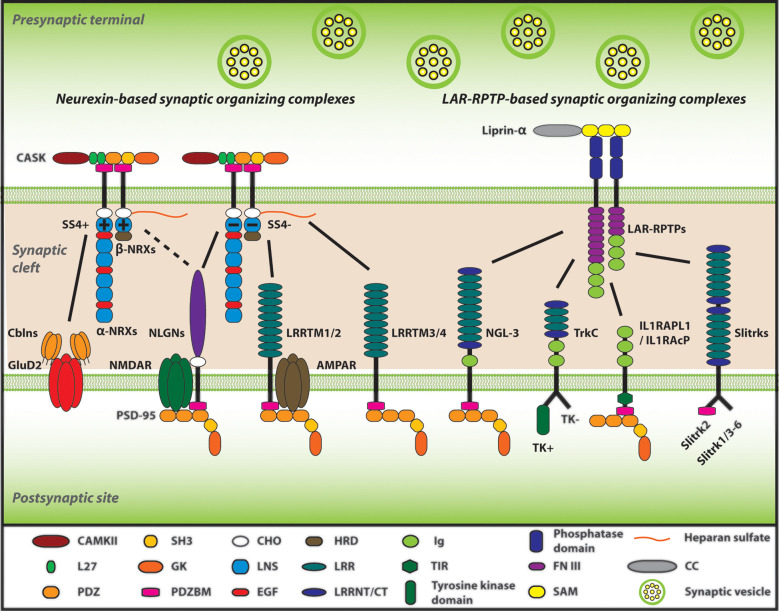
Neurexins (NRXs) and Leukocyte common antigen-related receptor protein tyrosine phosphatases (LAR-RPTPs) serve as presynaptic hubs to orchestrate synapse organization. The trans-interaction between pre- and post-synaptic organizers generate retrograde and anterograde “synaptogenic” signals through the synaptic cleft, resulting in the presynaptic organization, including synaptic vesicles clustering, and in the postsynaptic organization, encompassing the recruitment of neurotransmitter receptors [e.g., AMPA-type and NMDA-type glutamate receptor (AMPAR and NMDAR)] and scaffold proteins (e.g., PSD-95). As presynaptic molecular hubs, NRXs and LAR-RPTPs trans-synaptically interact with multiple specific postsynaptic organizers, for instance, NRXs-neuroligins (NLGNs), NRXs-leucine-rich-repeat transmembrane neuronal proteins (LRRTMs), PTPσ-neurotrophin receptor tropomyosin-related kinase C (TrkC), PTPσ/δ-Slit and Trk-like proteins (Slitrks), along with others. Furthermore, SS4 insertion modulates NRX interactome by regulating its binding properties with its diverse ligands. Solid lines indicate protein interactions, and the dashed line indicates that the insertion of SS4 in NRXs weakens NRX-NLGN interaction.

When neurons establish synaptic connections in the brain, many neuronal adhesion molecules mediate physical connections between axons and target neurons (Li and Sheng, 2003; Waites et al., 2005; Dalva et al., 2007). Importantly, a specific subset of the adhesion molecules has a further biological activity called “synaptogenic activity,” by which they promote pre- and/or post-synaptic organization to make synapses functional for neurotransmitter release and reception (Siddiqui and Craig, 2011; Missler et al., 2012). Such synaptogenic adhesion molecules have been called “synaptic organizers.” In general, their trans-synaptic complexes (herein called “synaptic organizing complexes”) drive bidirectional trans-cellular synaptogenic signals: (i) a retrograde signal from the target neuron to trigger the clustering of synaptic vesicles and assembly of the fusion apparatus on the axon; and (ii) an anterograde signal from the axon to trigger postsynaptic clustering of neurotransmitter receptors including α-amino-3-hydroxy-5-methyl-4-isoxazolepropionic acid-type glutamate receptors (AMPARs) and NMDARs and scaffolding molecules on the target neuron (Siddiqui and Craig, 2011; Missler et al., 2012). Such synaptogenic activities can be assessed by an artificial synapse formation assay based on co-culturing primary neurons with non-neuronal cells (e.g., COS-7 and HEK293 cells) transfected with the gene of interest (Craig et al., 2006). Numerous efforts over the years have identified and characterized many synaptic organizers, which can be grouped into two major categories; either neurexin (NRX)-based or Leukocyte common antigen-related receptor protein tyrosine phosphatases (LAR-RPTPs: composed of PTPσ, PTPδ as well as LAR)-based synaptic organizing complexes. NRXs and LAR-RPTPs act as presynaptic molecular hubs to trans-synaptically regulate synapse structure and function by making multiple trans-interactions with their specific postsynaptic organizers, such as NRX-neuroligin (NLGN), NRX-leucine-rich-repeat transmembrane neuronal proteins (LRRTMs), PTPσ-neurotrophin receptor tropomyosin-related kinase C (TrkC), PTPσ/δ-Slit and Trk-like proteins (Slitrks), LAR-netrin-G-ligand 3 (NGL3) and so on (Takahashi and Craig, 2013; Südhof, 2017; Figure 1). The most well-studied synaptic organizing complex is the NRX-NLGN complex, essential for synapse organization, transmission and plasticity as well as genetically linked with cognitive disorders such as autism spectrum disorders (ASD) and schizophrenia (Craig and Kang, 2007; Südhof, 2008, 2017; Kasem et al., 2018). Given the evidence of synaptic impairments in AD, recent studies have been trying to test whether and how Aβ interferes with synaptic organizers because of their pivotal roles in synapse physiology and cognitive function. Interestingly, our recent study has identified NRXs as a direct binding protein of AβOs (Naito et al., 2017b). Other groups have further uncovered the binding of Aβ with NLGN1 (Dinamarca et al., 2011; Brito-Moreira et al., 2017). This highlights the importance of studying the roles of synaptic organizers in the Aβ pathology of AD.

In this review, we first review the physiological synaptic roles of NRX-based and LAR-RPTP-based synaptic organizing complexes, which are closely relevant to AD synaptic pathology. We then review the emerging evidence of how synaptic organizers are involved in AD pathology, mainly focusing on Aβ pathology. Furthermore, considering their capability of Aβ binding (Dinamarca et al., 2011; Brito-Moreira et al., 2017; Naito et al., 2017b), we propose a new classification of synaptic organizers divided into two groups: Aβ-sensitive and Aβ-insensitive organizers, and discuss their implications in Aβ vulnerability and tolerance of synapses in AD. This would be essential to better understand the mechanisms involved in AD progression and give some insights into the development of novel therapeutic approaches for AD.

## Neurexin-Based Synaptic Organizing Complexes

### Neurexin

NRX is one of the most well understood presynaptic organizers. NRX is composed of six laminin/neurexin/sex-hormone-binding globulin (LNS) domains and three epidermal growth factor (EGF) domains, and additionally is one of the biggest genes existing in mammals (Missler and Südhof, 1998; Reissner et al., 2013). In mammals, NRX has three isoforms existing in different gene loci; *NRXN1*, *NRXN2*, and *NRXN3* (Südhof, 2008; Reissner et al., 2013). Moreover, each of these *NRXN* genes contains two alternative promoters leading to two different sizes: α-NRX, the longer isoform containing all six LNS domains, and β-NRX, the shorter isoform composed of only one LNS domain (identical to the sixth LNS domain of each α-NRX) as well as a unique short amino acid sequence at the N-terminal called histidine-rich domain (HRD; Südhof, 2008; Reissner et al., 2013). Using the common LNS domain, α/β-NRXs trans-synaptically interact with many postsynaptic organizers such as NLGNs and LRRTMs to act as a major presynaptic hub (Reissner et al., 2013; Südhof, 2017; Figure 1). The mRNA coding individual NRXs are broadly expressed in the brain in both overlapping and differential patterns (Uchigashima et al., 2019). For example, in the hippocampus, NRXN1/2/3α and 2β are highly expressed in all CA1/2/3 and dentate gyrus (DG) regions and NRXN1β is highly expressed in these regions except the CA1, although NRXN3β displays modulate and weak expression in the CA1–CA3 and DG, respectively (Uchigashima et al., 2019). At the synapse level, both α-NRXs and β-NRXs are thought to exist in both excitatory (glutamatergic) and inhibitory (GABAergic) synapses (Craig and Kang, 2007; Uchigashima et al., 2019). Although α-NRX has a higher expression level in comparison to β-NRX, β-NRX is more enriched at excitatory synapses (Neupert et al., 2015). Meanwhile, α-NRX and β-NRX expression levels have no significant difference at inhibitory synapses (Neupert et al., 2015). These suggest that both α-NRX and β-NRX play important roles at synapses. Artificial synapse formation assays have shown that β-NRXs can exert synaptogenic activity to induce postsynaptic organization for both excitatory and inhibitory synapses, whereas α-NRXs can induce postsynaptic organization for only inhibitory synapses, suggesting their different roles in synapse organization (Graf et al., 2004; Kang et al., 2008).

Triple-α-NRX knockout (KO) mice show a decrease in the neurotransmitter release from excitatory and inhibitory synapses by impairing presynaptic calcium channel function, despite reducing only inhibitory synapse number (Missler et al., 2003). Behavioral experiments on global KO mice for NRX1α or NRX2α have exhibited cognitive impairments similar to neurological symptoms of ASD and Schizophrenia (Etherton et al., 2009; Grayton et al., 2013; Dachtler et al., 2014, 2015). On the other hand, triple-β-NRX KO decreases excitatory synapse release probability *via* synaptic endocannabinoid signaling, leading to the impairment of presynaptic LTP and contextual memory (Anderson et al., 2015). These suggest that α/β-NRXs differently regulate synaptic functions and are indispensable for normal cognitive functions.

Also, each NRX has six alternative splicing sites (SS1-6) that regulate its binding properties with its binding partners (Tabuchi and Südhof, 2002; Treutlein et al., 2014; Südhof, 2017). Most of the studies on the NRX splicing sites so far have focused on addressing the roles of SS4. SS4 inclusion in NRX decreases its interaction with NLGN1 and loses its interaction with LRRTM2 (Koehnke et al., 2008; Ko et al., 2009a; Yamagata et al., 2018). On the other hand, at the parallel fiber-Purkinje cell synapses in the cerebellum, SS4 inclusion allows NRX to interact with cerebellin 1 (Cbln1) to make a triad complex with postsynaptic δ2 glutamate receptor (GluD2), which regulates the formation of this type of synapses and motor functions (Matsuda et al., 2010; Uemura et al., 2010). At the hippocampal CA1-subiculum synapses, SS4 insertion in NRX1 enhances NMDAR-mediated response, whereas SS4 insertion in NRX3 suppresses AMPAR-mediated response (Aoto et al., 2013; Dai et al., 2019). Thus, SS4 of NRX1 and NRX3 regulate different synaptic properties, even though NRX1 and NRX3 are supposed to largely share the same binding partners. Taken together, given the exceptional variety of NRX transcript variants expressed from three different genes with two independent promoters and six alternative splicings including SS4 (Reissner et al., 2013; Treutlein et al., 2014), the distinct roles of α/β-NRXs and those of NRX1/3 SS4 splicing have suggested that NRX variety may underlie the diversity and complexity of brain synaptic function and cognitive function.

### Neuroligin

NLGN has been well studied as one of the major NRX-interacting postsynaptic organizers (Bemben et al., 2015). NLGN has five subtypes: NLGN1-3, 4X, and 4Y (Bemben et al., 2015). In adult mouse brains, *NLGN1/2/3* mRNA is expressed in almost all neuronal populations with a different pattern, in which *NLGN2/3* mRNA expression is relatively higher than *NLGN1* in the brainstem, hypothalamus, and thalamus (Varoqueaux et al., 2006). In contrast, *NLGN4X* and *4Y* mRNA expression are very low in the human brain (Bolliger et al., 2001; Jamain et al., 2003). At the synapse level, NLGN1 and NLGN2 are mostly localized at excitatory and inhibitory synapses, respectively, whereas NLGN3 is localized at both excitatory and inhibitory synapses (Song et al., 1999; Varoqueaux et al., 2004; Budreck and Scheiffele, 2007). Artificial synapse formation assays have shown that NLGNs have a synaptogenic activity to induce presynaptic organization of excitatory and inhibitory synapses (Scheiffele et al., 2000; Graf et al., 2004; Chubykin et al., 2005; Craig et al., 2006; Naito et al., 2017b) through their trans-interaction with presynaptic NRXs (Ko et al., 2009b; Gokce and Südhof, 2013). Further, a recent study using NLGN1-4 conditional KO mouse brain slices with rescue experiments has shown that the NLGN1 extracellular domain, particularly its trans-interaction with presynaptic NRXs, is crucial for LTP (Wu et al., 2019). The extracellular domain of NLGN, mainly composed of acetylcholinesterase (ACE)-like domain, binds to the LNS6 domain of NRX in a calcium-dependent manner (Nguyen and Südhof, 1997; Südhof, 2008; Bemben et al., 2015). The ACE-like domain of NLGN contains an alternative splicing site that regulates their binding properties with NRXs, with exception to NLGN1 that has two alternative splicing sites (A and B; Chih et al., 2006; Ko et al., 2009a). Also, NLGN1 can form a complex with the major postsynaptic scaffold protein PSD-95 by its intracellular C-terminal tail, and this NLGN1-PSD-95 interaction is thought to be involved in postsynaptic molecular assembly (Irie et al., 1997). Indeed, NMDAR-mediated synaptic transmission is required for the intracellular domain of NLGN1 (Wu et al., 2019). On the other hand, the extracellular domain of NLGN1 also has a capability for postsynaptic recruitment of NMDARs, suggesting molecular and/or functional extracellular interaction between NLGN1 and NMDARs (Budreck et al., 2013). These extracellular and intercellular interactions of NLGN1 have been proposed to be the molecular basis underlying how NLGN1 is involved in synapse formation and function. Both *in vitro* and *in vivo* NLGN knockdown (KD) experiments result in a reduction of synapse number (Chih et al., 2005; Shipman et al., 2011; Shipman and Nicoll, 2012), while NLGN overexpression increases it (Prange et al., 2004; Boucard et al., 2005; Chih et al., 2005; Shipman et al., 2011). Also, NLGN1 KO shows LTP impairment in the hippocampus and spatial memory deficit (Blundell et al., 2010). On the other hand, NLGN1-3 triple KO impairs synapse transmission in both excitatory and inhibitory synapses without affecting their number (Chanda et al., 2017). Although the KD and KO studies show controversial results in synapse number, it is evident that NLGNs are crucial for synapse transmission and plasticity.

### LRRTM

LRRTM is another NRX-binding postsynaptic organizer. LRRTM family consists of LRRTM1-4 (Roppongi et al., 2017), which have distinct expression patterns in the brain (Laurén et al., 2003). LRRTM1/2 are highly expressed in all the layers of the cerebral cortex except layer 1, the granular layer in the hippocampal DG, and the hippocampal CA1-CA3 pyramidal layers (Laurén et al., 2003; Francks et al., 2007). LRRTM3/4 are highly expressed in the hippocampal DG, the cerebral cortex layer 2 and moderately expressed in the cerebral cortex layers 3–6 (Laurén et al., 2003). LRRTMs can promote the presynaptic organization of excitatory, but not inhibitory, synapses (Ko et al., 2009a; Linhoff et al., 2009; de Wit et al., 2013; Naito et al., 2017b). Interestingly, LRRTM1/2 bind to SS4-negative NRX [NRX SS4(−)], but not SS4-positive NRX [NRX SS4(+)], regardless of α- and β-NRX isoforms (Ko et al., 2009a; Siddiqui et al., 2010). Recently, it was reported that LRRTM3/4 bind to all NRX isoforms at the glycosylated region in the presence of heparan sulfate (HS; Roppongi et al., 2020). These NRX binding codes of LRRTMs may underlie the selective induction of excitatory, but not inhibitory, presynaptic organization by LRRTMs (Roppongi et al., 2017). Indeed, neuronal KD of LRRTM2 causes a significant reduction of excitatory synapses (de Wit et al., 2009). Also, LRRTM1/2 double KO mice show a selective reduction in AMPAR-mediated, but not NMDAR-mediated, synaptic transmission which leads to LTP impairment in hippocampal CA1 pyramidal neurons (Bhouri et al., 2018). This double KO mouse line also displays spatial memory impairment, suggesting that LRRTMs play a crucial role in memory formation by controlling synaptic transmission and plasticity (Bhouri et al., 2018).

## LAR-RPTP-Based Synaptic Organizing Complexes

### LAR/PTPσ/PTPδ

Besides NRX family members, LAR-RPTPs are the other major presynaptic organizers, consisting of LAR, PTPσ, and PTPδ (Takahashi and Craig, 2013). The mRNAs coding LAR, PTPσ and PTPδ are broadly expressed in various mouse brain areas in overlapping and differential patterns, for instance, in the hippocampal area, LAR is mainly expressed in the DG region, PTPσ is widely expressed in the CA1/2/3 as well as DG regions, and PTPδ is strongly expressed in the DG and the CA2 regions (Kwon et al., 2010). At synapse level, PTPσ is localized at excitatory, but not inhibitory, synaptic sites, whereas PTPδ is localized at inhibitory, rather than excitatory, synaptic sites (Takahashi et al., 2011; Han et al., 2018). According to artificial synapse formation assays, LAR, PTPσ, and PTPδ promote the postsynaptic organization of excitatory synapses, but not that of inhibitory synapses, as an anterograde synaptogenic signal (Woo et al., 2009; Takahashi et al., 2011; Yoshida et al., 2011). Also, as a retrograde synaptogenic signal, LAR, PTPσ and PTPδ mediate presynaptic organization of excitatory and/or inhibitory synapses induced by their postsynaptic binding partners (Woo et al., 2009; Takahashi et al., 2011, 2012; Yoshida et al., 2011; Han et al., 2018; Bomkamp et al., 2019). As a major presynaptic hub other than NRXs, LAR-RPTPs have capabilities to bind with many different postsynaptic binding partners such as TrkC, NGL3, Slitrk1-6, interleukin-1-receptor accessory protein-like 1 (IL1RAPL1) and interleukin-1 receptor accessory protein (IL1RAcP; Kwon et al., 2010; Takahashi et al., 2011, 2012; Yoshida et al., 2011, 2012; Takahashi and Craig, 2013; Um and Ko, 2013; Yim et al., 2013; Han et al., 2018).

Importantly, each of the LAR-RPTPs varies in their binding partner selectivity. For example, NGL3 binds to all the LAR-RPTPs, whereas TrkC binds to only PTPσ, and Slitrks bind to PTPσ/δ, but not LAR (Kwon et al., 2010; Takahashi et al., 2011; Yim et al., 2013). LAR-RPTPs are composed of three immunoglobulin (Ig) domains and eight or four Fibronectin III (FNIII) domains at the extracellular region, which are responsible for trans-synaptic interactions with the above-mentioned postsynaptic organizers (Takahashi and Craig, 2013; Um and Ko, 2013). Intracellularly, LAR-RPTPs bind to the scaffolding protein liprin-α to mediate presynaptic assembly (Dunah et al., 2005; Han et al., 2016a; Xie et al., 2020). These molecular interactions are essential for the anterograde and retrograde synaptogenic signals driven by the LAR-RPTP-based synaptic organizing complexes.

Previous KO mouse studies have revealed the importance of LAR-RPTPs for synaptic and cognitive function. Specifically, PTPσ KO decreases presynaptic release probability and NMDAR-dependent LTP in the hippocampal Schaffer-CA1 synapses and abnormally enhances novel object recognition (Horn et al., 2012; Han et al., 2020a; Kim et al., 2020). In contrast, a previous study by Uetani et al. (2000) showed that PTPδ KO increases release probability and LTP in the same type of synapses and impairs spatial learning and memory. Thus, PTPσ and PTPδ are indispensable for normal synaptic and cognitive functions in a distinct manner, which may be due to their different expression patterns and binding partners. To support this, mutations in PTPσ and PTPδ genes are associated with ASD and/or attention-deficit hyperactivity disorder (ADHD; Takahashi and Craig, 2013). Conversely, however, a more recent study has shown that PTPδ conditional KO does not affect release probability (Han et al., 2020b). Moreover, recent studies on conditional KO of all LAR-RPTPs have shown that they are involved in NMDAR-mediated synaptic transmission and LTP without affecting AMPAR-mediated transmission or synapse number (Sclip and Südhof, 2020). Further studies would be required to explain the apparent discrepancies and to more specifically address the synaptic roles of LAR-RPTPs.

### TrkC

TrkC is a member of the tropomyosin-receptor-kinase (Trk) family, which also includes TrkA and TrkB (Barbacid, 1994). The classical role of the Trk family is to recognize neurotrophins (NTs) such as NGF, BDNF, NT-3, and NT-4. TrkC is a specific receptor for NT-3, which promotes both neural crest cell proliferation and neuronal differentiation (Barbacid, 1994; Chao, 2003). TrkC mRNA is substantially expressed in the hippocampus and cortex of adult rat brains (Ringstedt et al., 1993), and TrkC protein is localized at excitatory, but not inhibitory, synapses in rat hippocampal neurons (Takahashi et al., 2011). Among the Trk family, only TrkC has a synaptogenic activity to selectively induce excitatory, but not inhibitory, the presynaptic organization as shown in artificial synapse formation assays and neuronal overexpression experiments (Takahashi et al., 2011; Naito et al., 2017b). While alternative splicing produces two subtypes of TrkC in terms of the presence or absence of an intracellular tyrosine kinase (TK) domain, both subtypes of TrkC contain an identical extracellular region composed of one LRR domain and two Ig domains (Valenzuela et al., 1993; Barbacid, 1994; Naito et al., 2017a). TrkC binds to PTPσ using the LRR and the first Ig domains (Takahashi et al., 2011; Coles et al., 2014) and binds to NT-3 using the second Ig domain (Urfer et al., 1995, 1998), suggesting distinct responsible domains for PTPσ- and NT3-binding and possible simultaneous binding of both PTPσ and NT3 to TrkC (Takahashi and Craig, 2013; Naito et al., 2017a), in which NT-3 may modulate a PTPσ-TrkC complex. To support this, recent studies including our own have revealed that NT-3 enhances the interaction between TrkC and PTPσ and the synaptogenic activity of TrkC, presumably through NT-3-induced dimerization of PTPσ-TrkC complexes (Ammendrup-Johnsen et al., 2015; Han et al., 2016b). Previous studies that characterized TrkC gene in transgenic or mutant mice also support the synaptic roles of TrkC and the involvement of TrkC in normal behaviors. For instance, a TrkC-overexpressing transgenic mouse line displays the elevated excitatory synaptic response in hippocampal CA1 as well as increased anxiety-like behavior and panic reaction (Dierssen et al., 2006; Sahún et al., 2007). Furthermore, TrkC KO mice show a decrease in hippocampal mossy fiber synapses as well as the impairment of synaptic maturation (Martínez et al., 1998; Otal et al., 2005).

### Slitrk

Slitrks have six isoforms found in three different chromosomes, and they are composed of two extracellular LRR domains at the extracellular region as well as a transmembrane and an intracellular domain that shares homology with Trks (Aruga and Mikoshiba, 2003; Aruga et al., 2003). An *in situ* hybridization study has shown different expression levels and patterns for each Slitrk isoform in the brain, especially high expression of Slitrk1/3/5 and moderate expression of Slitrk2/4 in the hippocampus and cortex of young mice (postnatal 10 days; Beaubien and Cloutier, 2009). Previous artificial synapse formation assays have shown that Slitrks have a unique synaptogenic activity, by which Slitrk1/2 induce both excitatory and inhibitory presynaptic organization *via* presynaptic PTPσ and PTPδ, respectively, while Slitrk3 selectively induces inhibitory, but not excitatory, presynaptic organization *via* presynaptic PTPδ (Takahashi et al., 2012; Yim et al., 2013). The study characterizing Slitrk3 KO mice has further supported selective involvement of Slitrk3 in inhibitory synapse development (Takahashi et al., 2012) by detecting a decrease in inhibitory synapse number and function as well as seizure behaviors. On the other hand, RNAi-based knockdown studies as well as neuronal overexpression ones have indicated selective involvement of Slitrk1/2 in excitatory synapse number and function (Yim et al., 2013; Schroeder et al., 2018; Han et al., 2019). Also, Slitrk1 KO mice exhibit elevated anxiety behaviors (Katayama et al., 2010), and Slitrk5 KO mice display obsessive-compulsive–like behaviors with decreases in glutamate receptors and excitatory synaptic transmission in cortico-striatum synapses (Shmelkov et al., 2010). Together, each Slitrk isoform plays a distinct role in organizing excitatory or inhibitory synapses for normal cognitive functions.

## Synaptic Organizers in AD

Considering the above-mentioned crucial roles of synaptic organizers in physiological synaptic functions, they are expected to be also substantially involved in synaptic dysfunction in AD. Indeed, we have recently uncovered that NRXs interact with AβOs and that this interaction impairs normal trafficking of NRXs on axon surface as well as excitatory presynaptic organization induced by NRX-binding partners such as NLGN1/2 and LRRTM2 (Naito et al., 2017b). Furthermore, given our artificial synapse formation data and cell surface Aβ binding data, we propose a new classification of synaptic organizers into two groups with regards to Aβ pathology: Aβ-sensitive and Aβ-insensitive synaptic organizers as discussed below.

## Aβ-Sensitive Synaptic Organizers in AD

### Neurexin: A Novel Binding Partner of Aβ Oligomers

**Figure 2:**
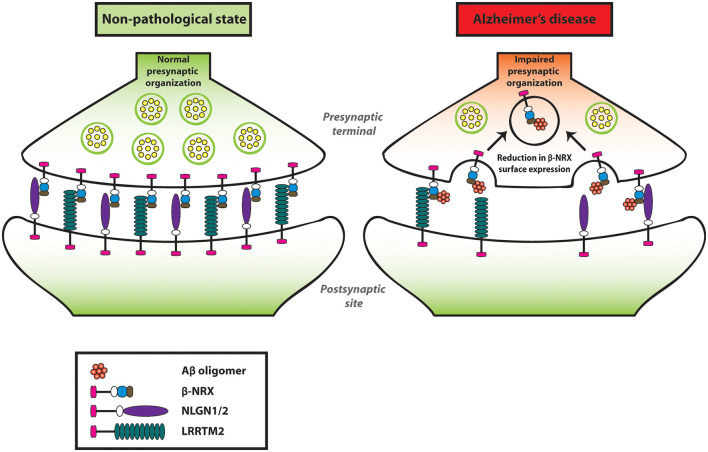
Amyloid-β oligomers (AβOs) impair presynaptic organization by reducing β-NRXs on the axon surface. AβOs bind to the histidine-rich domain (HRD) of β-NRXs. This interaction leads to the reduction of β-NRX surface expression on the axons without interfering with its ability to bind to NLGN1 or LRRTM2. By reducing the surface level of β-NRXs, presumably through enhanced endocytosis, AβOs impair NLGN1/2- and LRRTM2-mediated presynaptic organization.

Our group has performed an *in situ* binding assay screen using a non-physiological concentration of AβOs (250 nM, monomer equivalent) to identify synaptic organizers that interact with AβOs. Out of the 19 synaptic organizers that we tested, interestingly, only NRXs were isolated (Naito et al., 2017b). Similarly, another group has also reported that NRX1α and NRX2α bind to AβOs (Brito-Moreira et al., 2017) by performing a plate binding assay using recombinant proteins of NRX1α and NRX2α with AβOs. Our group has further performed a domain analysis and identified that the HRDs of all β-NRXs are responsible for AβO binding (Naito et al., 2017b). Moreover, the oligomeric but not the monomeric form of Aβ has an interaction with NRX1β. Interestingly, the interaction of AβOs with NRX1β does not interfere with its ability to bind to its synaptic partners such as NLGN1 or LRRTM2 (Naito et al., 2017b). To further clarify the AβO influence on β-NRX function in the neurons, we quantified the cell surface expression level of NRX1β on axons by performing time-lapse imaging of NRX1β extracellularly tagged with a pH-sensitive GFP (SEP-NRX1β; Mahon, 2011) transfected in hippocampal primary neurons before and after AβO treatment (Naito et al., 2017b). Interestingly, AβO treatment reduces surface expression of NRX1β on the axons (Naito et al., 2017b). However, SEP-NRX1β lacking the HRD is not affected by AβOs, suggesting that AβOs trigger cell surface reduction of NRX1β by binding to its HRD (Naito et al., 2017b). Currently, the physiological role of HRD in β-NRXs is not well understood, therefore it should be addressed for a better understanding of Aβ-induced synaptic pathology. Taken together, AβOs interact with β-NRXs in an HRD-dependent manner, and this interaction reduces β-NRX expression on the axon surface, presumably through enhanced endocytosis, leading to an impairment in NRX-mediated presynaptic assembly (Figure 2). Further, interestingly, β-NRX conditional triple KO increases tonic endocannabinoid signaling, such as the tonic activation of cannabinoid receptor type 1 (CB1R), to impair excitatory synaptic transmission and LTP (Anderson et al., 2015). Therefore, it is also possible that AβO-induced β-NRX surface reduction may enhance tonic endocannabinoid signaling for synaptic impairment. Indeed, it has been reported that CB1R activity is enhanced in the anterior thalamus in an AD mouse model named 3XTg-AD (Manuel et al., 2016; Basavarajappa et al., 2017). Moreover, the synaptic phenotypes of the β-NRX triple KO are detected in burst-firing, but not regular-firing, subiculum neurons, indicating synapse specificity of β-NRXs at the cellular level. Therefore, it would be interesting to elucidate whether and how Aβ affects the β-NRX-mediated endocannabinoid signaling and the synaptic specificity of β-NRXs in AD.

We also identified that the SS4 of both the α and β isoforms of NRX1/2 are responsible for AβO binding (Naito et al., 2017b). However, the role of AβO binding to the SS4 sites of NRX1/2 remains to be elucidated. Our time-lapse imaging has suggested no effect of the AβO binding to NRX1β SS4 site on NRX1β expression on axon surface (Naito et al., 2017b), suggesting that it may play a different role from the HRD of NRX1β. Given that the SS4 insertion of presynaptic NRX1 increases postsynaptic NMDAR responses and thereby enhances NMDAR-dependent LTP at the hippocampal CA1-subiculum synapses (Dai et al., 2019), it is likely that the AβO binding to NRX1 SS4 site could impact NMDAR-dependent LTP, which is impaired by AβO treatment and in AD model mouse lines with Aβ overproduction (Wang et al., 2004; Hwang et al., 2017; Liu et al., 2019). On the other hand, the SS4 insertion of presynaptic NRX2 does not affect either NMDAR or AMPAR responses in the subiculum. Further studies on NRX2 SS4 as well as NRX1 SS4 are necessary to elucidate their physiological synaptic roles and involvement in Aβ synaptic pathology.

In addition to synaptic dysfunction, AβO binding to NRX could potentially play other roles in Aβ pathology, such as Aβ oligomer formation as an Aβ nucleation factor and/or neuronal AβO uptake as an Aβ receptor. To determine whether NRX can accelerate Aβ oligomerization as well as fibrillar aggregation, the thioflavin T fluorescence assay (Xue et al., 2017), in which Aβ monomers are incubated with/without NRX recombinant proteins, would be useful in further studies. Furthermore, to test whether and how NRXs are involved in neuronal uptake of AβOs, it would be worthy to perform live-cell imaging of NRX KO/KD neurons or control neurons treated with AβOs tagged with pH-sensitive dye [e.g., pHrodo (Han and Burgess, 2010; Mao et al., 2016)] that allows imaging of only internalized AβOs.

Due to the toxic and dysfunctional effects of AD pathology on neurons, the expression level of many genes including NRXs is altered in AD patients compared to healthy controls. A recent study has reported the differentially-expressed genes (DEG) in AD patients’ brains based on published microarray data sets. Interestingly, *NRXN3* gene expression is significantly decreased and has the second-highest DEG in AD patients. Moreover, in the hippocampus, *NRXN3* gene expression is decreased in both AD- and aging-related groups (Zheng et al., 2018). Similarly, we have reported that synaptosome fractions of J20 mice [Alzheimer’s model mice overproducing Aβ (Mucke et al., 2000)] have a significant reduction in β-NRXs as well as a reduction trend in α-NRXs, compared to their wild-type littermates (Naito et al., 2017b). These reports suggest that the expression levels of NRXs in AD are downregulated. However, it is not fully understood which of the NRX isoforms are mainly affected in AD and which brain regions in AD display changes in NRX expression. Therefore, *in situ* hybridization for each NRX isoform in AD model mice could provide us with a better understanding of how AD pathology affects NRX-mediated synapses.

### Neuroligin: Aβ-Induced Synaptogenic Dysfunction and a Role as Aβ Deposition Stabilizer

Our artificial synapse formation assay has shown that AβO treatment significantly diminishes excitatory, but not inhibitory, presynaptic organization induced by NLGN1 and NLGN2 (Naito et al., 2017b). Given that AβO treatment reduces surface expression of NRX1β on axons, but has no effect on NRX1β-NLGN1 interaction, Aβ impairment of NLGN1-induced presynaptic organization may be due to decreased amount of axonal β-NRXs rather than direct interference with β-NRX-NLGN1 interaction (Naito et al., 2017b; Figure 2). While the artificial synapse formation assay is thus useful to determine Aβ sensitivity of NLGNs by assessing the effect of AβOs on the formation of NRX-NLGN-based synapses, it is also crucial to investigate their effect on the maintenance of NRX-NLGN-based synapses for better understanding of Aβ synaptic pathology. To address this, additional research needs to be carried out by performing artificial synapse formation assays with AβO treatment after synapses have been formed by NLGN-expressing fibroblasts.

A recent study has shown that NRXs are modified with heparan sulfate (HS) and that the synaptogenic activity of NLGN1/2 requires their interaction with the NRX HS chains as well as their protein domain-based NRX interaction (Zhang et al., 2018). Although it remains to be tested whether and how Aβ pathology and NRX HS modification are involved with each other, it has been shown that Aβ can directly interact with HS chains and HS core proteins (Buée et al., 1993; Watson et al., 1997; Cui et al., 2013). Furthermore, neuronal HS deficiency suppresses Aβ deposit in the brain of AD model mice (Liu et al., 2016), suggesting a physical and functional interaction between Aβ and HS-modified proteins, which presumably could include NRXs. Given that NRX HS modification does not affect NRX surface trafficking itself (Zhang et al., 2018), Aβ sensitivity of NRX-NLGN1/2 complexes might depend on not only the Aβ-impaired NRX trafficking on axon surface but also NRX HS modification level in AD condition.

**Figure 3:**
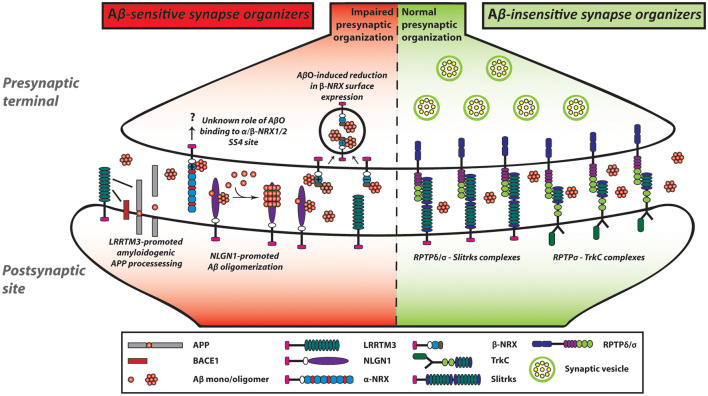
NRX-based and LAR-RPTP-based synaptic organizing complexes display contrasting Aβ sensitivity. AβOs exert a pathological influence on NRX-based synaptic organizing complexes. In contrast, LAR-RPTPs and their post-synaptic binding partners appear to be resistant to AβO-induced deleterious effects on synapses. AβOs bind to the HRD of β-NRXs as well as to the SS4 of NRX1/2. Furthermore, AβOs reduce the expression of β-NRXs on axon surface in an HRD-dependent but SS4-independent manner. However, the role of the NRX SS4 in Aβ synaptic pathology remains to be understood. In addition to NRXs, NLGN1 can interact with Aβ and act as an Aβ deposition stabilizer to accelerate Aβ oligomer formation and fibrillar aggregation. Although AβOs affect not only NRXs but also LRRTMs and NLGNs, we propose that AβO-mediated β-NRX surface reduction may account the most in Aβ-impaired synapse organization. As an additional role of LRRTMs in the Aβ pathway, LRRTM3 interacts with amyloid precursor protein (APP) as well as β-site APP cleaving enzyme 1 (BACE1) and promotes amyloidogenic APP processing to enhance Aβ production. Thus, NRXs and their partners are highly linked with Aβ pathology, which may result in Aβ vulnerability of synapses in Alzheimer’s disease (AD). In contrast, AβOs do not interact with LAR-RPTPs or their postsynaptic partners including TrkC and Slitrks. Accordingly, LAR-RPTP-based complexes may contribute to making synapses more tolerant of Aβ pathology in AD. Solid lines indicate protein interactions.

Although our group performed *in situ* binding assays and concluded that AβOs did not interact with NLGN1 (total four different splicing isoforms), NLGN2 or NLGN3 (Naito et al., 2017b), two independent groups have reported that AβOs interact with at least NLGN1 (Dinamarca et al., 2011; Brito-Moreira et al., 2017). To demonstrate the AβO-NLGN1 interaction, one group performed a plate binding assay using NLGN1 recombinant proteins and AβOs (Brito-Moreira et al., 2017), and the other group used fluorescence spectroscopy to monitor Aβ-induced quenching of intrinsic tryptophan fluorescence from NLGN1 because of the prevalence of tryptophan amino acids in NLGN1, while not at all present in Aβ (Dinamarca et al., 2011). The discrepancy between our results and theirs might come from the sensitivity of the experimental methods. In this sense, a plate binding assay and fluorescence spectroscopy may have higher sensitivity than the *in situ* binding assays we performed. Also, their study using a thioflavin T fluorescence assay and electron microscopy have suggested that NLGN1 plays a role as a nucleating factor on Aβ aggregation, ultimately facilitating Aβ oligomer formation at the excitatory postsynaptic sites (Dinamarca et al., 2011; Figure 3). Given the three previously described pieces of evidence: (1) NRXs, as well as NLGN1, interact with AβOs; (2) NRXs trans-synaptically interact with NLGNs; and (3) NLGN1 is localized at excitatory synapses, it would be interesting to test whether and how NRXs regulate Aβ aggregation process together with NLGN1 at excitatory synapses as synaptic Aβ nucleating factors.

### LRRTM: Aβ-Induced Synaptogenic Dysfunction and a Role in APP Processing

Like the case of NLGN1/2, the synaptogenic activity of LRRTM2 is sensitive to AβOs (Naito et al., 2017b). Specifically, our artificial synapse formation assay has shown that AβO treatment significantly decreases LRRTM2-induced excitatory presynaptic organization in cultured hippocampal neurons. On the other hand, AβO treatment does not affect NRX1β-LRRTM2 binding. Given that AβO treatment reduces the surface expression level of NRXs on the axons (Naito et al., 2017b) and that LRRTM1/2 share same NRX binding code (Siddiqui et al., 2010), AβOs are supposed to dampen the synaptogenic activity of LRRTM1/2 by decreasing the amount of cell surface NRXs on axons (Naito et al., 2017b). More specifically, the AβO-mediated LRRTM1/2 dysfunction may be due to the reduction of axonal expression of β-NRX SS4(−) rather than α-NRX SS4(−) or α/β-NRX SS4(+) for the two reasons: LRRTM1/2 selectively bind to α/β-NRX SS4(−; Siddiqui et al., 2010); and AβOs do not bind to α-NRX SS4(−; Naito et al., 2017b). These suggest that β-NRX SS4(−) may be a key determinant for Aβ sensitivity of LRRTM1/2-mediated excitatory synapses. Importantly, the J20 AD model mouse line shows a more significant reduction in synaptic expression of β-NRX than that of α-NRX (Naito et al., 2017b). Therefore, it would be worthy to analyze the expression of β-NRX SS4(−) and SS4(+) separately and that of LRRTM1/2 in AD animal models and/or in AD patients’ brain for better understanding of Aβ vulnerability of excitatory synapses *in vivo* condition.

In contrast to LRRTM1/2, LRRTM3/4 bind to all NRX isoforms including NRX1γ, which lacks the LNS domain (Roppongi et al., 2020). So far, no study has tested whether and how AβOs affect the synaptogenic activity of LRRTM3/4, and given that NRX-LRRTM3/4 interaction requires the NRX HS modification, but not the LNS domain (Roppongi et al., 2020), investigating this matter would be helpful to understand how AβOs physically and functionally interact with NRX HS chain.

In addition to the synaptogenic role of LRRTM3, a previous study using a siRNA screen has identified LRRTM3 as a positive modulator of APP processing (Majercak et al., 2006). The siRNA-based LRRTM3 knockdown in SH-SY5Y human neuroblastoma cells reduces Aβ secretion and the production of the intracellular C-terminal fragments (CTFs) by β-secretase (βCTF), suggesting that LRRTM3 positively modulates BACE1 processing of APP (Figure 3). Indeed, LRRTM3 overexpression increases Aβ secretion. A follow-up study has further shown that LRRTM3 interacts with both APP and BACE1 and that LRRTM3 is colocalized with APP in cultured cortical neurons from the Tg2576 AD model mice (Lincoln et al., 2013). On the contrary, another group has reported that LRRTM3 KO in the AD model mouse does not alter the Aβ production, suggesting that LRRTM3 may not be an essential regulator of Aβ production *in vivo* (Laakso et al., 2012). The authors have pointed out that one possibility for this discrepancy is that LRRTM4, which is the closest paralog of LRRTM3, could compensate for the Aβ production. While the underlying mechanism and the synaptic role of the LRRTM3-dependent modulation of APP processing need to be addressed, these findings suggest that NRX-LRRTM3-mediated synapses may be vulnerable to Aβ due to local Aβ overproduction by LRRTM3 as well as Aβ binding to NRXs at the synapse level.

## Influence of Aβ-Insensitive Synaptic Organizers in AD

In addition to the identification of NRXs and their binding partners as Aβ-sensitive synaptic organizers, our recent study has illustrated the potential presence of Aβ-insensitive synaptic organizers (Naito et al., 2017b). The *in situ* binding screens have demonstrated that except NRXs, the other tested synaptic organizers including LAR-RPTPs and their binding partners, such as TrkC and Slitrk1-6, show no significant binding of AβOs (Figure 3). Consistent with the binding results, AβO treatment does not affect the synaptogenic activity of TrkC and Slitrk2 to induce excitatory presynaptic organization, which is mediated by PTPσ and/or PTPδ (Naito et al., 2017b; Figure 3). Therefore, the LAR-RPTPs and their binding partners could be classified as Aβ-insensitive synaptic organizers. In line with this, a previous postmortem study (Connor et al., 1996) has shown that the expression level of TrkC is unchanged in the hippocampus of AD patients. Specifically, TrkC immunostaining remains high in the granular as well as the pyramidal layers in the hippocampus in both AD and healthy control samples. These suggest that even during AD progression, TrkC may contribute to synapse maintenance by positively regulating synaptic tolerance to Aβ through its Aβ-resistant trans-synaptic bridge with PTPσ. Indeed, some synapses are preserved even at the late stage of AD (Scheff, 2003). To better understand the molecular mechanisms underlying the structural and functional preservation of synapses in AD and the possible correlation between LAR-RPTP-based synaptic organizing complexes and synaptic tolerance to Aβ, it would be important to investigate the expression levels of LAR-RPTPs and their postsynaptic partners in AD brains.

To further validate whether Aβ-insensitive synaptic organizers such as LAR-RPTPs and TrkC have a protective role against Aβ in AD synapses, it would be worth testing whether their KO in AD model mouse brain accelerates synaptic pathology and/or if their overexpression in AD model mouse brain decelerates synaptic pathology. Such studies will be essential to validate the roles of Aβ-insensitive synaptic organizers in Aβ tolerance of synapses and can potentially be approached as a therapeutic strategy.

Moreover, another postmortem study has shown that the expression level of NT-3, a TrkC neurotrophic ligand, is comparable between AD patients and healthy controls in any of the brain regions, although a slight non-significant decrease in NT-3 is detected in the cortex (Durany et al., 2000). Given that NT-3 enhances PTPσ-TrkC interaction and their synaptogenic activity (Ammendrup-Johnsen et al., 2015; Han et al., 2016b), it has also been suggested that for synapse maintenance in AD, NT-3 might reinforce PTPσ-TrkC complex to increase synaptic tolerance to Aβ.

## The Role of Synaptic Organizers in Tau Pathology

Besides Aβ pathology, tau pathology is the other major AD hallmark. While there have been very few studies on the involvement of synaptic organizers in tau pathology, one study has reported the involvement of NLGN1 and LRRTM2 in cell-to-cell propagation of tau pathology (Calafate et al., 2015). When NLGN1- or LRRTM2-transfected HEK293 cells are co-cultured with hippocampal neurons expressing human mutant P301L tau, which leads to aggressive tau aggregation, the transfected HEK293 cells enhance tau aggregation in the co-cultured neurons, suggesting that NLGN1 and LRRTM2 mediate cell-to-neuron tau pathology propagation. Moreover, according to tau propagation assays using microfluidic culture devices, neuron-to-neuron propagation of tau pathology *via* synaptic connections is decreased by NLGN1 KD. Thus, tau propagation between neurons could be facilitated by synaptic connections mediated by synaptic organizing complexes such as NRX-NLGN1 and NRX-LRRTM2.

Given the previous studies showing that Aβ triggers and/or enhances tau pathology (Götz et al., 2004; Bennett et al., 2017; Lee et al., 2017), it would also be interesting to test whether and how AβO binding to NRX influences tau pathology in AD. Notably, NRXs bind to a scaffolding protein called CASK (Hata et al., 1996; LaConte et al., 2016), and the phosphorylation and membrane distribution of CASK are regulated by cyclin-dependent kinase 5 (CDK5), a key player that up-regulates tau hyper-phosphorylation and thereby leads to NFT (Lee and Tsai, 2003; Samuels et al., 2007; Shukla et al., 2012). Furthermore, CASK has been reported as one of the up-regulated biomarkers in the hippocampus of AD patients (Gómez Ravetti et al., 2010). This evidence gives rise to the interesting possibility that NRX might play a role in Aβ-induced tau pathology *via* CASK/CDK5.

## Influence of Other Aβ-Sensitive Cell Adhesion Molecules

Some cell adhesion molecules other than the canonical synapse organizers have also been reported to interact with and be affected by Aβ, such as EphB2 and NCAM2. EphB2 is an ephrin B2 receptor that is localized at the postsynaptic site. A previous study has shown that Aβ interacts with EphB2, reducing the expression of surface and total EphB2 due to enhanced EphB2 degradation, ultimately leading to NMDAR-mediated LTP impairment (Cissé et al., 2011). Similarly, Aβ binds to NCAM2 and reduces NCAM2 expression levels in cultured hippocampal synaptosome (Leshchyns’ka et al., 2015). Also, Aβ affects the number of AMPAR subunit GluRA1-containing glutamatergic synapses in an NCAM2-dependent manner (Leshchyns’ka et al., 2015). Thus, some cell adhesion molecules exhibit Aβ sensitivity and would contribute to further weakening trans-synaptic cell adhesions in AD.

## Conclusion and Future Directions

A growing number of studies are accumulating on the roles of synaptic organizers in AD pathology. Among the many different synaptic organizers, it is possible to classify them into two groups with regards to Aβ pathology; Aβ-sensitive and Aβ-insensitive synaptic organizers. Specifically, β-NRX directly binds to AβOs, and this interaction reduces β-NRX expression on axon surface (Naito et al., 2017b), suggesting that β-NRX is a major Aβ-sensitive synaptic organizer. However, given the discrepancy among the studies regarding Aβ binding to NLGN1 (Dinamarca et al., 2011; Brito-Moreira et al., 2017; Naito et al., 2017b), it is also important to confirm whether Aβ-insensitive synaptic organizers including LAR-RPTPs have no Aβ-binding ability by performing multiple independent experimental approaches. Given that NRX-based synaptic organizing complexes are essential for regulating synapse organization, synaptic transmission and synaptic plasticity under physiological conditions and are also required for normal cognitive functions (Südhof, 2017; Kasem et al., 2018), the Aβ-induced dysregulation/dysfunction of NRXs would be a key mechanism underlying synaptic pathology and cognitive decline in AD. On the other hand, Aβ-insensitive synaptic organizers, such as LAR-RPTPs, may contribute to synapse maintenance and preservation in AD and/or compensate for the dysfunctions of Aβ-sensitive synaptic organizers since Aβ-sensitive and Aβ-insensitive synaptic organizers are linked with each other *via* intracellular protein interactions based on liprin-α (Pulido et al., 1995; Wei et al., 2011; Takahashi and Craig, 2013; LaConte et al., 2016) and share some roles in synapse organization and functions. Together, the Aβ-based classification of synaptic organizers would be useful for a better understanding of the molecular basis determining Aβ vulnerability and tolerance of synapses in AD brains. Since the role of synapse organizers in Aβ binding is still an emerging field, current research has been limited to *in vitro* studies. Therefore, it will be essential that future studies address the *in vivo* roles of Aβ binding of synapse organizers to better classify them with regards to Aβ sensitivity and characterize their involvement in AD.

Given no effects of AβOs on inhibitory presynaptic organization induced by NLGN1/2 (Naito et al., 2017b), this review mainly focuses on the roles of synaptic organizers in Aβ impairment of glutamatergic excitatory synapses. However, Aβ also diminishes GABAergic inhibitory synaptic transmission by enhancing GABA_A_ receptor endocytosis (Ulrich, 2015). Given that some synaptic organizers such as NLGN2 and Slitrk3 preferentially regulate inhibitory synapse organization (Poulopoulos et al., 2009; Takahashi et al., 2012; Li et al., 2017), further studies would be also necessary to address whether and how synaptic organizers are involved in Aβ-induced dysfunction of inhibitory synapses and dysregulation of GABA_A_ receptors.

Considering how this evidences regarding synaptic organizers can be translated into AD therapy, we propose that the modification of their Aβ sensitivity to make synapses less vulnerable and/or more tolerant to Aβ would be an interesting and potential approach for alleviating AD synaptic pathology. To modify the Aβ sensitivity, the AβO binding mode of HRD of β-NRX, NRX1/2 SS4, and NLGN1 should be elucidated. This would help predict and screen small molecules and peptides that block AβO-NRX and AβO-NLGN1 interactions and could consequently make synapses less vulnerable to Aβ. Also, determining the amino acid residues responsible for NRX and NLGN1 binding to AβO may allow us to generate Aβ-resistant NRXs and NLGN1 mutants, which might be useful for developing new gene therapeutic approaches to ameliorate Aβ pathology in neuron culture, AD animal models and hopefully in AD patients. On the other hand, to make synapses more tolerant to Aβ, the up-regulation and/or functional enhancement of Aβ-insensitive synaptic organizers, such as TrkC, could be a potential for alternative therapeutic approaches. As mentioned above, NT-3 has been identified as not only TrkC ligand in the canonical neurotrophin pathway (Barbacid, 1994; Chao, 2003) but also a synaptogenic enhancer of PTPσ-TrkC complex for excitatory synapse organization (Ammendrup-Johnsen et al., 2015; Han et al., 2016b; Naito et al., 2017a). Notably, a previous *in vitro* study has shown that NT-3 application on primary cortical neurons protects them from Aβ-induced toxicity (Lesné et al., 2005). Further studies should be carried out on TrkC and/or NT-3 up-regulation in AD mouse models to validate their beneficial effects on Aβ synaptic pathology *in vivo*. Thus, targeted manipulation of Aβ sensitivity of synaptic organizers should have great potential in developing novel therapeutic strategies for AD.

## Author Contributions

AL and HK structured and wrote the manuscript. NC made figures and wrote figure legends. HT gave structural and contextual input and revised the manuscript.

## Conflict of Interest

The authors declare that the research was conducted in the absence of any commercial or financial relationships that could be construed as a potential conflict of interest.
